# The *JAK2* V617F mutation in isolated neutropenia

**DOI:** 10.17179/excli2017-941

**Published:** 2018-01-02

**Authors:** Stephen E. Langabeer

**Affiliations:** 1 Cancer Molecular Diagnostics, St. James's Hospital, Dublin, Ireland

## ⁯⁯

Dear Editor,

Neutropenia, defined as a sustained neutrophil count of less than 1.5 x 10^9^/L, is a common hematological abnormality that can be either transient (causes include infection, drugs or immune mediated) or chronic (causes include extrinsic factors such as nutritional and immune factors, or intrinsic defects such as bone marrow failure syndromes or specific neutropenic syndromes) (Newburger and Dale, 2013[4]). Identification of the *JAK2* V617F mutation is a major diagnostic criterion for the classical myeloproliferative neoplasms (MPN) of polycythemia vera, essential thrombocythemia and primary myelofibrosis. However, this mutation is also observed in other myeloid malignancies such as acute myeloid leukemia and myelodysplastic syndrome/MPN (MDS/MPN) with ring sideroblasts and thrombocytosis, albeit at a considerably lower frequency than that of the classical MPN. Apart from MDS, in which diagnosis is based on number of cytopenias, morphologically dysplastic lineages, presence of ringed sideroblasts, peripheral blood and bone marrow blast counts, and cytogenetics (Arber et al., 2016[1]) neutropenia is not a diagnostic criterion for other myeloid malignancies. Despite this and the absence of a requirement for excluding *JAK2* V617F in diagnostic algorithms for neutropenia (Gong et al., 2013[3]; Palmblad et al., 2014[5]) an isolated neutropenia anecdotally appears to be a continual, if infrequent trigger for requesting *JAK2* V617F mutation analysis.

A retrospective audit was therefore performed in order to address the value of screening for the *JAK2 *V617F mutation in patients presenting with neutropenia. In an eleven and a half year period from January 2006 to June 2017 inclusive, 15,627 diagnostic requests were received for *JAK2* V617F identification at a molecular diagnostic centre for hematological malignancies. Clinical details of neutropenia were provided on 278 requests. Of these 278 requests, 45 (16.2 %) had details of isolated neutropenia whereas the remaining 233 (83.8 %) had in addition, at least one further clinical feature noted including anemia, thrombocytopenia, bone marrow fibrosis, splenomegaly or hepatosplenomegaly, increased lactate dehydrogenase, or a leucoerythroblastic blood picture. The methodology for detection of the *JAK2* V617F mutation was unchanged throughout the audit period. The *JAK2* V617F was not detected in any of the patients with clinical details of isolated neutropenia but was detected in 14 of the 233 (6.0 %) patients with additional clinical details suggestive of a myeloid malignancy.

While the number of patients analysed with an isolated neutropenia remains modest, this audit suggests that testing for the *JAK2* V617F mutation is not warranted in patients with neutropenia unless accompanied by additional features suggestive of a myeloid malignancy. Acknowledging evolution to MDS has been observed in a minority of patients with chronic neutropenia (Fattizzo et al., 2015[2]) prolonged follow up is required with targeted molecular analysis necessary when clinically indicated.

## Conflict of interest

The author declares no conflict of interest.
